# In silico analyses of metagenomes from human atherosclerotic plaque samples

**DOI:** 10.1186/s40168-015-0100-y

**Published:** 2015-09-03

**Authors:** Suparna Mitra, Daniela I. Drautz-Moses, Morten Alhede, Myat T. Maw, Yang Liu, Rikky W. Purbojati, Zhei H. Yap, Kavita K. Kushwaha, Alexandra G. Gheorghe, Thomas Bjarnsholt, Gorm M. Hansen, Henrik H. Sillesen, Hans P. Hougen, Peter R. Hansen, Liang Yang, Tim Tolker-Nielsen, Stephan C. Schuster, Michael Givskov

**Affiliations:** Singapore Centre on Environmental Life Sciences Engineering (SCELSE), Nanyang Technological University, Singapore, Singapore; Norwich Medical School, University of East Anglia, Norwich, UK; Institute of Food Research, Norwich Research Park, Norwich, UK; The Department of Forensic Medicine, Faculty of Health Sciences, University of Copenhagen, Copenhagen, Denmark; Costerton Biofilm Center, Department of Immunology and Microbiology, University of Copenhagen, Copenhagen, Denmark; Department of Vascular Surgery RK, Rigshospitalet, University of Copenhagen, Copenhagen, Denmark; Department of Clinical Microbiology, Rigshospitalet, Copenhagen, Denmark; Department of Cardiology, Gentofte University Hospital, Copenhagen, Denmark

## Abstract

**Background:**

Through several observational and mechanistic studies, microbial infection is known to promote cardiovascular disease. Direct infection of the vessel wall, along with the cardiovascular risk factors, is hypothesized to play a key role in the atherogenesis by promoting an inflammatory response leading to endothelial dysfunction and generating a proatherogenic and prothrombotic environment ultimately leading to clinical manifestations of cardiovascular disease, e.g., acute myocardial infarction or stroke. There are many reports of microbial DNA isolation and even a few studies of viable microbes isolated from human atherosclerotic vessels. However, high-resolution investigation of microbial infectious agents from human vessels that may contribute to atherosclerosis is very limited. In spite of the progress in recent sequencing technologies, analyzing host-associated metagenomes remain a challenge.

**Results:**

To investigate microbiome diversity within human atherosclerotic tissue samples, we employed high-throughput metagenomic analysis on: (1) atherosclerotic plaques obtained from a group of patients who underwent endarterectomy due to recent transient cerebral ischemia or stroke. (2) Presumed stabile atherosclerotic plaques obtained from autopsy from a control group of patients who all died from causes not related to cardiovascular disease. Our data provides evidence that suggest a wide range of microbial agents in atherosclerotic plaques, and an intriguing new observation that shows these microbiota displayed differences between symptomatic and asymptomatic plaques as judged from the taxonomic profiles in these two groups of patients. Additionally, functional annotations reveal significant differences in basic metabolic and disease pathway signatures between these groups.

**Conclusions:**

We demonstrate the feasibility of novel high-resolution techniques aimed at identification and characterization of microbial genomes in human atherosclerotic tissue samples. Our analysis suggests that distinct groups of microbial agents might play different roles during the development of atherosclerotic plaques. These findings may serve as a reference point for future studies in this area of research.

**Electronic supplementary material:**

The online version of this article (doi:10.1186/s40168-015-0100-y) contains supplementary material, which is available to authorized users.

## Background

Cardiovascular disease is the leading cause of death worldwide, and atherosclerosis, its primary cause, is a chronic inflammatory disease traditionally associated with risk factors such as male sex, age, smoking, hypertension, hyperlipidemia, obesity, and diabetes. However, elevated circulating levels of inflammatory markers, e.g., high-sensitivity C-reactive proteins are also risk markers for atherosclerotic disease and increasing evidence indicates that infections and chronic inflammatory diseases, e.g., rheumatoid arthritis, are also linked with increased risk of atherosclerosis [1–3]. Indeed, direct microbial infection in the vessel wall can promote inflammatory responses and generation of a proatherogenic and prothrombotic environment, while secreted bacterial products, e.g., lipopolysaccharide and heat shock proteins, can also indirectly induce autoimmunity and other immunoinflammatory mechanisms that may contribute to the atherosclerotic process [1, 2].

Specifically, a number of infectious agents have been suggested to promote atherosclerosis, e.g., *Chlamydia pneumoniae*, *Helicobacter pylori*, Hepatitis C virus, *Pseudomonas aeruginosa*, and *Cytomegalovirus* and oral bacteria such as *Porphyromonas gingivalis*, *Aggregatibacter actinomycetemcomitans*, and *Prevotella intermedia* involved in marginal periodontitis, have been associated with atherosclerotic disease, e.g., acute myocardial infarction [4, 5]. Indeed, previous studies have identified DNA from a broad variety of bacteria in atherosclerotic plaques [6]. Bacterial 16S rDNA signatures from environmental microorganisms and several nosocomial pathogens and viruses were recently found in atherosclerotic lesions of patients with coronary heart disease [7, 8]. As compared to asymptomatic stable plaques, culprit lesions associated with atherosclerotic disease manifestations, e.g., myocardial infarction or stroke, are associated with increased inflammation and thrombosis, but differences in bacterial profiles of unstable vs. stable atherosclerotic plaques have not been investigated.

Even though the above pathogens have been reported to be present in plaques, there is a lack of published high-resolution investigations of dynamic composition of microbial infectious agents that may be associated with development of atherosclerosis. The traditional approach for identifying microbes from target sites such as culturing and 16S rRNA PCR sequencing are quite limited for characterization of numerous infectious agents [4, 6]. Recent progress in deep sequencing technology has provided another potential approach for high throughput and comparative analysis of the atherosclerosis-associated microbial agents. Also, metagenomics has the potential to characterize microbial communities in various sites of human body and give a deeper understanding of their impact on human physiology and diseases [9]. However, many challenges still remain in applying metagenomics analysis of atherosclerotic plaque samples. For example, atherosclerotic plaques contain large amounts of human genome DNA which can reduce the microbial genome DNA reads during sequencing. Similar considerations apply for analyses of target tissue samples from other host inflammation-associated diseases like periodontal diseases, non-healing ulcers, or traumatic wounds.

Here, with the use of two different types of atherosclerotic tissue samples, symptomatic atherosclerotic plaques from patients with symptomatic atherosclerotic disease and asymptomatic atherosclerotic plaques from patients that have atherosclerotic tissues died from other causes than atherosclerosis, we performed a comparative metagenomic analysis of the contribution of microbial community to the development of atherosclerotic disease. The atherosclerotic microbial communities were analyzed by applying deep sequencing and a multistage approach using different methods to reduce human genomic reads and hereby achieve improved understanding of the bacterial communities potentially involved in human disease. We identified bacterial families of potential importance for eliciting the transition from asymptomatic to symptomatic atherosclerotic disease.

## Results

### Sequencing and data processing

All 22 arterial plaque samples resulted in 2,610,268,774 reads. After mapping these reads against Hg19 using bowtie 2, the average amount of non-Hg19 reads is 884,727,044 (average 33.89 % per sample, Table 1). These non-Hg19 reads were aligned on NCBI-nr and imported in MEGAN using parameters as detailed in the “Methods” section.

**Table 1:** Sample statistics and read assignments

Patients	Platform	Sample ID-DNA	Raw reads	Non-Hg19 reads	Non-Hg19 (%)	RapSearch processed (as in MEGAN)	Assigned in MEGAN (MSc:50 MSp25 MinCompl:0.44 and paired protocol)	Bacteria	% Bacteria
Cases	Hiseq 2000	48	93,124,682	31,504,036	33.83	8,404,029	3,763,197	243,336	2.90 %
	Hiseq 2000	49	101,840,018	53,068,718	52.11	12,405,108	6,772,304	436,463	3.52 %
	Hiseq 2000	50	94,765,328	43,109,046	45.49	11,643,905	6,419,975	505,239	4.34 %
	Hiseq 2000	51	112,426,390	50,653,838	45.06	10,711,699	4,899,567	216,511	2.02 %
	Hiseq 2000	52	88,328,470	36,795,952	41.66	10,574,678	5,766,665	492,013	4.65 %
	Hiseq 2000	53	124,000,764	41,858,084	33.76	10,057,354	4,676,002	243,775	2.42 %
	Hiseq 2000	54	93,124,682	82,366,266	88.45	19,199,623	9,229,157	365,993	1.91 %
	Hiseq 2000	237	141,334,092	58,853,792	41.64	6,946,473	3,854,397	249,369	3.59 %
	Hiseq 2000	238	153,302,968	36,976,828	24.12	5,216,154	3,002,923	835,412	16.02 %
	Hiseq 2000	239	154,652,444	48,177,200	31.15	5,643,923	3,045,461	222,363	3.94 %
	Hiseq 2000	240	101,591,496	46,000,276	45.28	5,166,877	2,640,033	110,186	2.13 %
	Hiseq 2000	241	99,927,824	42,032,952	42.06	4,607,355	2,471,259	153,425	3.33 %
	Hiseq 2000	242	82,850,664	46,721,284	56.39	5,002,916	2,716,756	135,617	2.71 %
	Hiseq 2000	243	104,094,892	52,853,464	50.77	5,726,930	3,095,542	193,819	3.38 %
	Hiseq 2500	977 (P0613)	111,699,176	13,191,406	11.81	2,344,220	955,602	74,646	3.18 %
	Miseq V1	P0613 repeat	7,184,184	980,004	13.64	759,262	228,262	31,889	4.20 %
		977 + APD1	118,883,360	14,171,410	11.92	3,103,482	1,183,864	106,535	3.43 %
Controls	Hiseq 2000	55	105,779,932	29,019,712	27.43	8,911,853	4,040,359	293,160	3.29 %
	Hiseq 2000	56	128,471,814	10,767,822	8.38	4,680,735	1,922,688	204,397	4.37 %
	Hiseq 2000	232	127,173,774	25,793,632	20.72	3,267,969	1,726,714	164,711	5.04 %
	Hiseq 2000	233	166,547,592	29,282,304	17.58	4,559,468	2,634,338	649,226	14.24 %
	Hiseq 2000	234	114,673,124	37,683,568	32.86	4,550,457	2,477,740	193,659	4.26 %
	Hiseq 2000	235	151,195,284	34,767,160	22.99	4,633,661	2,550,975	231,926	5.01 %
	Hiseq 2000	236	152,179,180	32,269,700	21.21	4,254,572	2,319,403	204,778	4.81 %
Total			2,610,268,774	685,143,146	26.25				4.60 %

Details of sample information, sequencing read statistics, and assignments of reads after different stages of data processing

### Taxonomic annotation

**Fig. 1:**
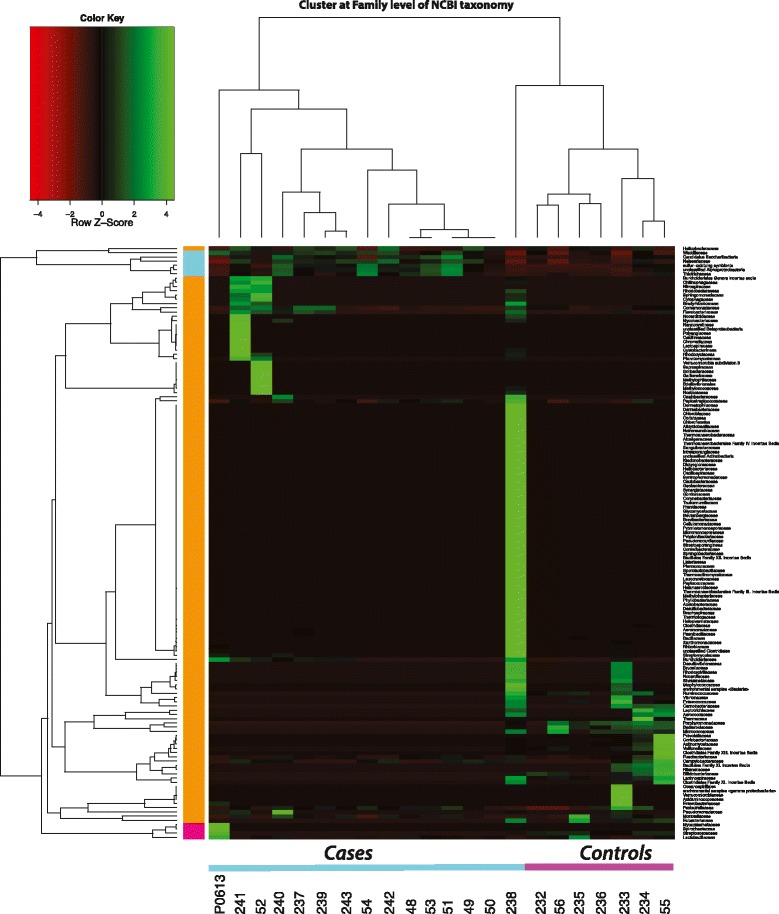
Taxonomic comparison of all DNA samples. Hierarchical clustering result of “family” level taxonomic comparisons of data from unstable atherosclerotic plaques from 15 patients with symptomatic atherosclerotic disease (unstable plaques) and stable plaques from a control group of 7 patients that died from other causes than atherosclerosis (controls). *Red* indicates down-regulation, *green* indicates up-regulation, and *black* indicates no change in read abundance level comparing to all samples. Hierarchical clustering was computed with average linkage, whereas Pearson correlation was used for clustering the families (*rows*) and Spearman correlation was used for clustering the datasets (*columns*), respectively

For all non-Hg19 annotated reads, 2 to 16 % (mean 4.6 %) were assigned as bacteria in different samples. The rest of reads were Eukaryota. Table 1 provides details of sequencing read statistics and assignments of reads after different stages of data processing. Figure 1 depicts a hierarchical clustering result of family level taxonomic comparison data for all 22 samples. Interestingly, samples 238 and P0613 were mostly different, and among the other samples, all unstable plaques clustered together, apart from all stable plaque controls that clustered separately. Interestingly, the asymptomatic atherosclerotic plaques have more abundance of host microbiome-associated microbial families such as *Porphyromonadaceae*, *Bacteroidaceae*, *Micrococcaceae*, and *Streptococcaceae* than the symptomatic atherosclerotic plaques (Fig. 1). In contrast, the symptomatic atherosclerotic plaques have more abundance of pathogenic microbial families such as *Helicobacteraceae*, *Neisseriaceae*, and sulfur-consuming families such as sulfur-oxidizing symbionts and *Thiotrichaceae* than the asymptomatic atherosclerotic plaques (Fig. 1).

Additional file 1: Figure S1 provides a multiple comparison tree view using taxonomic annotation of all 12 samples at “family” level of NCBI taxonomy. In this figure, the samples from patients with symptomatic atherosclerosis (unstable plaques) are displayed in white and the control samples of stable lesions (are displayed in gray color).

Despite our efforts to clean up human gene sequences from the samples, we found that some of the human-like sequences remained detectable. This was probably caused by the high level of variation in our samples compared to the hg19 reference genome. Indeed, some human sequences can cause false hits to species which have similar sequence structure. Some of the species hits that are likely to be of human origin were those for *Microbacterium laenivormans*, *Cyanothece*, *Coprobacillus*, and *Aster Yellows Phytoplasma* which were therefore marked with black crosses in Additional file 1: Figure S1, and we discarded these four species before clustering (Fig. 1).

**Fig. 2:**
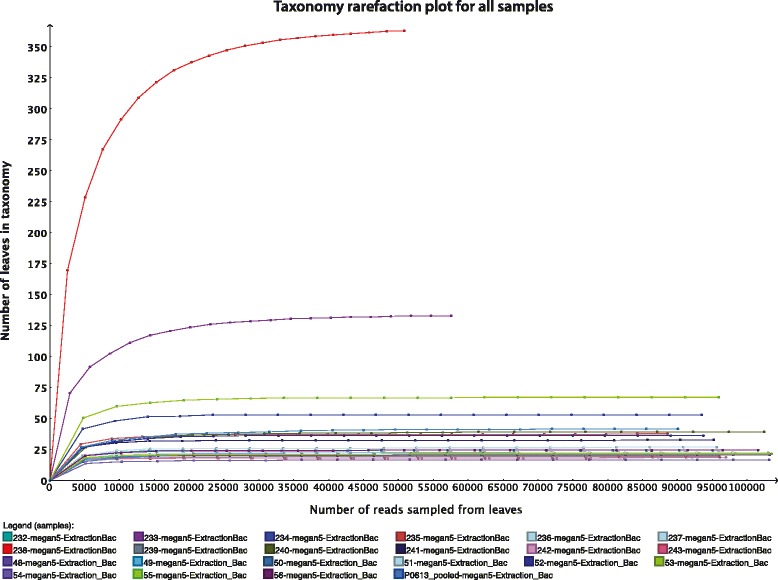
Rarefaction. Rarefaction plot using annotated species profile for all 22 (unstable and stable) atherosclerotic plaque samples. These curves show the number of nodes that would be present if based on 10, 20, and up to 90 % of the reads

**Fig. 3:**
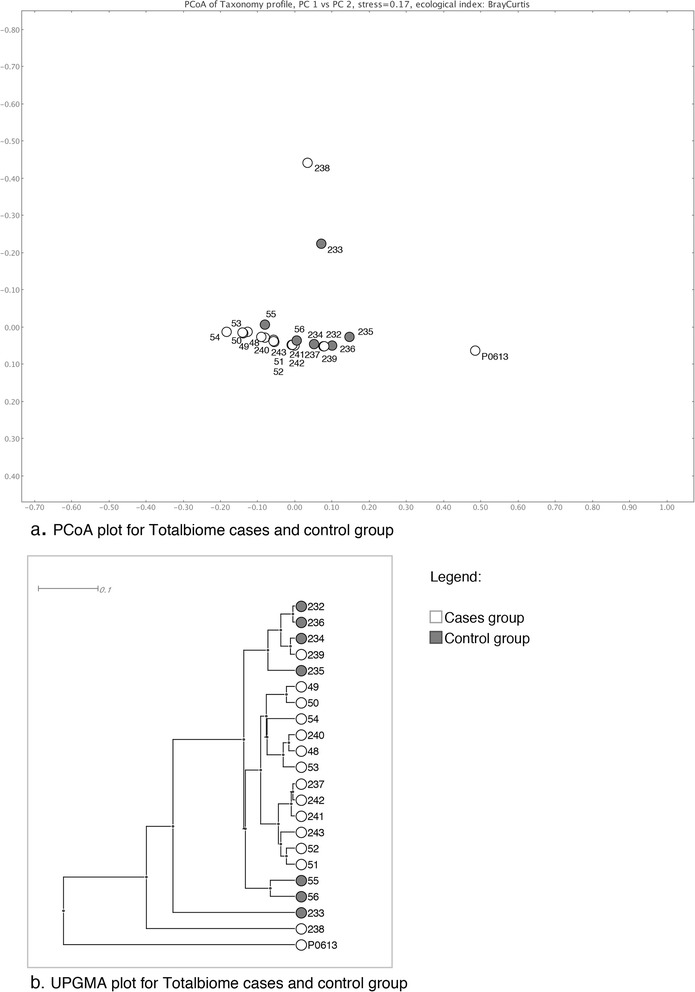
Multiple comparison clustering. Clustering of the 22 atherosclerotic samples using **a** principal coordinate analyses (PCoA) and **b** unweighted pair group method with arithmetic mean (UPGMA) tree. The 15 unstable atherosclerotic plaque samples (cases) are displayed in *white* and the 7 stable plaque samples (controls) in *gray*

**Fig. 4:**
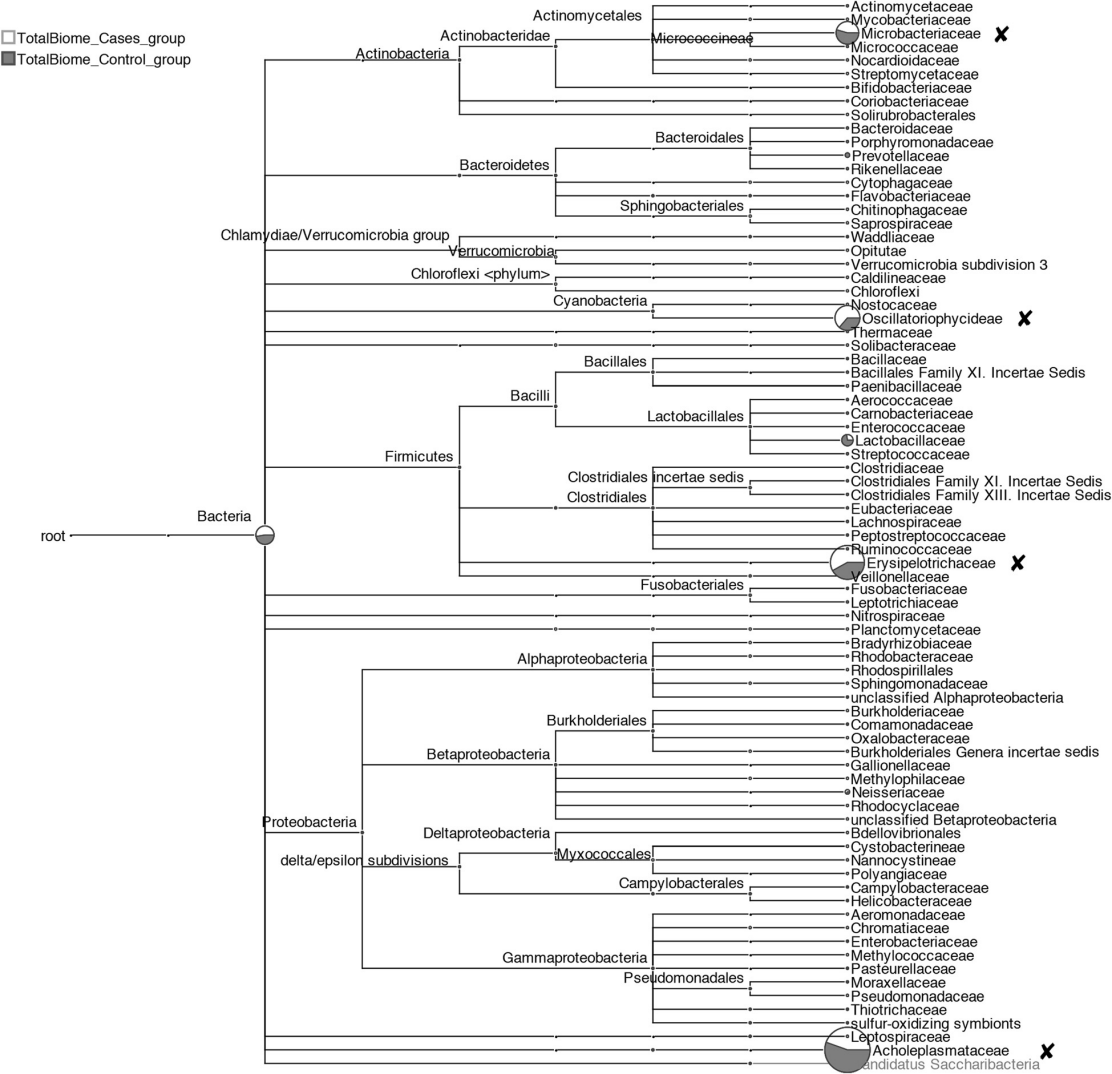
Taxonomic comparison of all DNA samples. Comparative tree view of merged samples (excluding samples 233, 238, and P0613) from unstable atherosclerotic plaques (*white*) and control group of stable plaques (*gray*) at “family” level of NCBI taxonomy. The scale shows the log value of reads assigned directly to a particular node. Some of the species hits that are likely to be of human origin are marked with *black crosses*

**Fig. 5:**
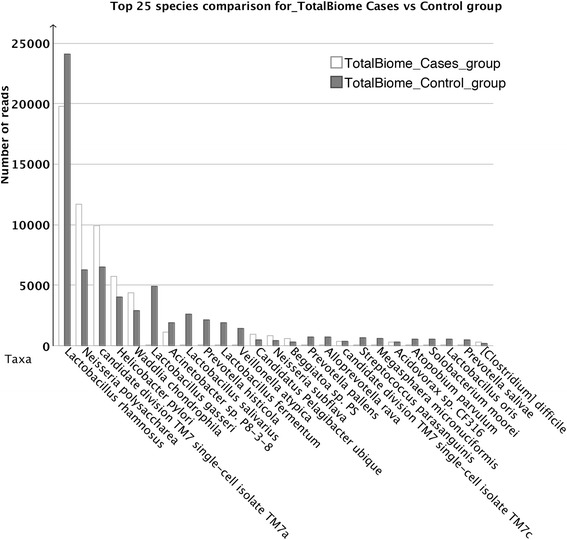
Top 25 abundant species. Top 25 abundant species for all (unstable and stable) atherosclerotic plaque samples (excluding samples 233, 238 and P0613) are displayed in a bar chart

From sequence statistics (Table 1) and the rarefaction curve (Fig. 2), it is apparent that 2 (sample 233 and 238) of the 22 samples had much higher sequencing depth than the other samples (Table 3). Additionally, the PCoA plot and UPGMA tree of the taxonomic profile (Fig. 3) showed clustering among the unstable plaques and among the control samples, respectively, using species level data. Only three samples (233, 238, and P0613) remained separated, and it appears that the high diversity caused by the sequencing depth is a major contributor for separation of samples 233 and 238. For P0613, the species profile appeared very different from all other samples (Fig. 1). We therefore omitted these three samples from the merged samples. Figure 4 depicts a multiple comparison tree view at “family” level of NCBI taxonomy using the normalized total biome for the merged samples. The taxonomic profile in this tree view was consistent with results of comparisons between all samples (Additional file 1: Figure S1). Figure 5 shows the 25 most abundant species in the total biome for merged samples (excluding 4 biased species, as mentioned earlier). Among the most prevalent species in case samples were *Lactobacillus rhamnosus* MTCC 5462, *Neisseria polysaccharea* ATCC 43768, *H. pylori* Hp P-4, and *Acidovorax* spp. CF316.

### Functional analyses and comparison of total biome

MEGAN’s functional analyses using SEED and KEGG classification are shown in Additional file 2: Figure S2 and Additional file 3: Figure S3 at the second level of the SEED and KEGG hierarchy. From both figures, it is apparent that all of the involved metabolisms were driven mostly by samples 233 (violet) and 238 (red), and this might also be caused by higher sequencing depth for this two samples as described in the “Taxonomic annotation” section.

**Fig. 6:**
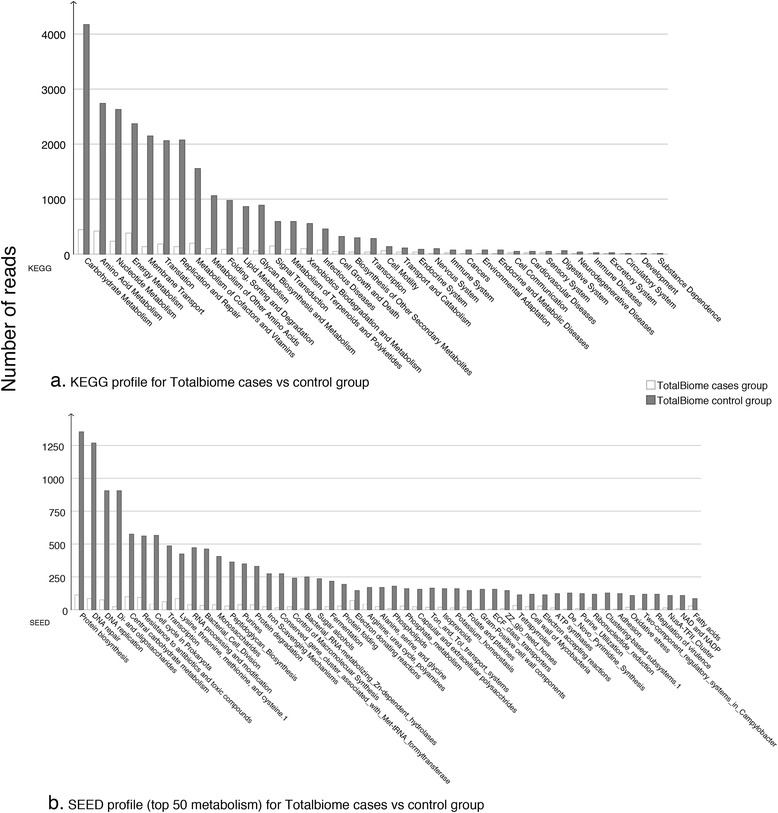
Functional comparison of total biome. Differences between total biome for unstable atherosclerotic plaque samples (*white*) and stable plaque samples (*blue*) are displayed with comparative bar charts. **a** depicts the difference of KEGG orthologies (level 2) and **b** provides comparison of the two groups with the top 50 SEED-level 2 subsystems

Additionally, from functional annotation of the total biome for the two groups of samples (unstable vs. stable plaques, excluding samples 233, 238, and P0613), we noticed significant differences in basic metabolic and disease pathway signatures. Based on normalized comparisons of total bacterial reads in two groups, reads assigned to functional metabolism were much lower for unstable plaques (white) compared to stable lesions (gray). Figure 6a depicts the difference of KEGG orthologies (level 2), and Fig. 6b provides comparison of the top 50 SEED-level 2 subsystems for the two sample groups. These functional annotations suggested involvement of basic metabolic and disease pathways such as those related to “carbohydrate”, “amino acid”, and “energy” metabolisms. Moreover, entities perceived to be of predominant relevance to atherosclerosis, i.e., “cardiovascular diseases”, “circulatory system”, “immune system”, and “cell growth and death”, and to “infectious diseases”, respectively, are also found.

**Fig. 7:**
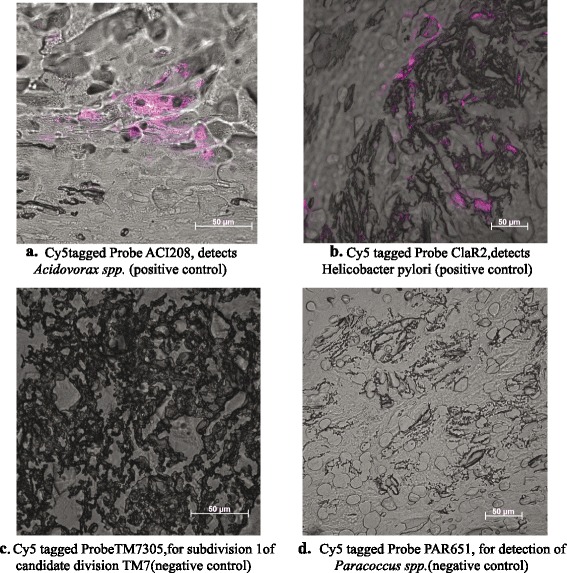
FISH images of *Acidovorax* spp*.* and *H. pylori* in atherosclerotic plaque samples. Representative results of plaque samples after applying the *Acidovorax* spp*.-*specific probe (**a**), *H. pylori*-specific probe (**b**), as well as two negative control probes, i.e., *Candidate division TM7* (**c**), and *Paracoccus* spp. (**d**), on atherosclerotic plaque material from several different patients. We observed morphogically distinct clusters of *Acidovorax* spp*.* and *H. pylori* cells surrounded by atherosclerotic tissue material

To characterize the growth physiology of potential bacterial species in the plaque, we performed FISH analysis to visualize the distribution of two selected species from the most abundant bacterial species, *Acidovorax* spp. and *H. pylori*, within multiple plaque samples (see the “Methods” section). Table 3 provides the details of the probes. Figure 7 shows representative images of plaque samples after applying the *Acidovorax* spp.-specific probe, *H. pylori*-specific probe, as well as two negative control probes (*Candidate division TM7* and *Paracoccus* spp.) on plaque material from several different patients. We observed morphologically distinct clusters of *Acidovorax* spp*.* and *H. pylori* cells surrounded by atherosclerotic tissue material (Fig. 7a, b). In contrast, the two negative control probes produced almost no signal after being applied to plaque samples (Fig. 7c, d).

## Discussion

Atherosclerosis is a chronic inflammatory disease that is generally perceived to be driven by classical risk factors (smoking, diabetes, hypertension, etc.). However, evidence accumulated in the last 25 years suggests that infection can also play an important contributory role by direct and indirect mechanisms [1–3]. In the present study, we used deep sequencing and metagenomic analysis to provide a high-resolution investigation of microbial species in atherosclerotic plaques from stable asymptomatic lesions and unstable lesions removed at surgery. We provided details of a multi-step protocol to reduce human DNA reads in order to obtain a comprehensive picture of the microbial community present in the diseased arterial wall. A similar protocol can be applied to any host-associated metagenomics study.

To date, a wide range of infectious agents have been linked to atherosclerosis, and while *H. pylori*, *Cytomegalovirus*, Hepatitis C virus, and other species have also been implicated, the current weight of evidence has arguably favored the involvement of *C. pneumoniae* and periodontal organisms such as *P. gingivalis* [2–4]. However, these works were based on final point sampling of the symptomatic atherosclerotic plaques samples. The lack of asymptomatic atherosclerotic plaques samples limits our understanding of the roles of microbial agents for contributing to the development of the atherosclerotic diseases. Also, recent research has suggested that the contribution from an aggregate “infectious” burden induced by a large number of pathogens is much more important for atherosclerosis development than any single organism [3, 10, 11].

Our work provides the first comparative metagenomic analysis of atherosclerotic plagues from the symptomatic atherosclerotic disease patients and asymptomatic atherosclerotic samples from a control group of patients that have atherosclerotic tissues died from other causes than atherosclerosis. The asymptomatic atherosclerotic plaques have more abundance of host microbiome-associated microbial families such as *Porphyromonadaceae*, *Bacteroidaceae*, *Micrococcaceae*, and *Streptococcaceae* [12] than the symptomatic atherosclerotic plaques (Fig. 1). This result suggests that these host microbiome-associated microbial families are one of the first colonizers of the arteries. These early colonizers might support the growth of sulfur-consuming families such as sulfur-oxidizing symbionts and *Thiotrichaceae* [13] and pathogens such as *Helicobacteraceae* and *Neisseriaceae*, which were found to be abundant in the symptomatic atherosclerotic plaques (Fig. 1). It is well known that homocysteine, an intermediate in sulfur-containing amino acid methionine metabolism, is highly related to the vascular disease, and arteriosclerosis patients have elevated levels of homocysteine in their blood [14]. The elevated levels of homocysteine might facilitate the thrive of sulfur-consuming families. The presence of pathogens might enhance the degeneration of elastic fibers and fragmentation of the internal elastic membrane by their elastase activity. Further study is required to investigate the potential interactions of commensal and pathogen populations during the transition from asymptomatic atherosclerotic plaques to symptomatic atherosclerotic plaques.

Some of the infectious agents that are most prevalent in our symptomatic atherosclerotic samples, e.g., *Acinetobacter*, *Acidovorax*, and *N. polysaccharea* have not been reported previously. FISH observation validated the presence of biofilm-like structures of these pathogens in the symptomatic atherosclerotic plague samples (Fig. 7). In addition, the presence of multiple pathogens in atherosclerotic plaques and their potential organization in biofilms may be part of the explanation underlying other pertinent findings in this area of research, e.g., absence of effect of antibiotic treatment on clinical endpoints in high-risk patients with coronary artery disease, and an apparent correlation between oral, gut, and atherosclerotic plaque microbiotas [5, 15, 16]. Indeed, a very recent study suggested the presence of *P. aeruginosa* biofilms within the plaque in patients with advanced atherosclerotic disease [5]. In our study, however, we found only a very low number of *Pseudomonas* reads in four symptomatic atherosclerotic plague samples (average 0.08 % reads of all assigned bacteria) out of 15 unstable plaque samples and in one asymptomatic atherosclerotic plague samples sample (0.048 % reads) among seven plaques, respectively. These differences in observation of *Pseudomonas* abundance between their study and our study might due to the fact that we used deep shotgun sequencing approach, which might have helped to obtain greater picture of the microbial community. More studies are clearly needed to determine the putative identity, organization, and role of bacteria detected in atherosclerotic plaques.

The functional analyses reported in the current study represent an innovative method to investigate mechanisms of potential relevance to atherosclerosis. Interestingly, these results show basic (carbohydrate, amino acid, and energy metabolism) pathways, whereas potential pathways for causing cardiovascular diseases and infectious diseases are also identified. However, these results should be viewed as preliminary and hypothesis-generating, and more research is required to provide more insights into atherosclerosis pathobiology. In addition to this context, it is worth to mention that currently, we are working on the next phase of this project using transcriptomics. RNA will help us in better understanding of activity of these metabolisms.

Important limitations to this study include lack of true negative controls, i.e., arterial samples without atherosclerosis, and absence of histologic examinations to establish signs of plaque (in)stability. Although we found an abundance of microbial genetic material, we cannot conclude that these agents were viable within the atheromatous plaque. For example, bacterial DNA found within the sampled atheroma could represent DNA fragments from bacteria engulfed and killed elsewhere in the body by phagocytic cells that subsequently entered the atherosclerotic vessel segment. Study limitations also include risk of microbial contamination of tissue samples.

## Conclusions

This paper describes a novel approach to apply high-throughput sequencing and whole-genome shotgun metagenomics on plaque tissue samples to investigate microbiome diversity of the plaque obtained from patients with a thrombotic event and controls with non-symptomatic plaques. Our data provides evidence that suggest a wide range of microbial agents in atherosclerotic plaques and an intriguing new observation that shows these microbiota displayed differences between symptomatic and asymptomatic plaques as judged from the taxonomic profiles in these two groups of patients. Additionally, functional annotations reveal significant differences in basic metabolic and disease pathway signatures between these groups.

## Methods

### Patients and samples

For this study, we used atherosclerotic tissue samples from a group of 15 patients that underwent elective carotid endarterectomy following repeated transient ischemic attacks or minor strokes (samples from symptomatic atherosclerotic plaques as cases (Table 1). Our methods and experimental manuals were approved by The National Committee on Health Research Ethics (Danish) and was granted by the Ethical Committee of the region of Copenhagen (H-3-2011-013). Further, we have asymptomatic atherosclerotic plaques from seven persons who died from causes not related to atherosclerotic disease (samples from stable plaques as controls; Table 1) that originated from the tissue bank at the Department of Forensic Medicine (Approval No. 1501230). The approvals do not allow us to collect and use patient/person data for the analysis. Consequently, after collection, all 22 samples were treated in a blinded fashion during lab process, sequencing, and data analyses.

### Sample collection and DNA sequencing

Appropriate material from diseased arteries taken at sites of macroscopic atherothrombotic plaque was surgically dissected. Cross-sectional pieces of tissue 3–5-mm thick were prepared and immediately stored at −80 °C.

DNA was extracted using QIAGEN’s DNeasy Blood & Tissue kit, following the “Purification of Total DNA from Animal Tissues (Spin-Column)” protocol. Briefly, tissue was cut into tiny pieces of no more than 25 mg on a clean Petri dish on dry ice and then homogenized in ATL buffer with a 5-mm stainless steel bead (QIAGEN) on a Tissuelyser at 50 Hz for 2 min or until tissue was completely homogenized. After homogenization, proteinase K was added and the sample was incubated at 56 °C until it was completely lysed. The remaining steps of the protocol were performed according to QIAGEN’s recommendation. DNA was eluted with 100 μL of buffer AE.

Prior to library preparation, the quality of the DNA samples was assessed on a Bioanalyzer 2100, using a DNA 12000 Chip (Agilent). Sample quantitation was performed using Quant-iT™ PicoGreen ® dsDNA Reagent.

Next-generation sequencing library preparation was prepared by following Illumina’s TruSeq DNA Sample Preparation protocol. The samples were sheared on a Covaris S220 to ~500 bp, following the manufacturer’s recommendation. Size selection was performed on a Sage Science Pippin Prep instrument using a 1.5 % EtBr agarose cassette and selecting for a tight peak around 620 bp. Each library was uniquely tagged with one of Illumina’s TruSeq LT DNA barcodes to allow library pooling for sequencing.

Library quantitation was performed using Invitrogen’s PicoGreen assay and the average library size was determined by running the libraries on a Bioanalyzer DNA 7500 chip (Agilent). Library concentrations were normalized to 4 nM and validated by qPCR on a StepOne Plus real-time thermocycler (Applied Biosystems), using qPCR primers recommended in Illumina’s qPCR protocol, and Illumina’s PhiX control library as standard. Libraries were then pooled and sequenced in two lanes of an Illumina HiSeq2000 sequencing run at a read length of 101 bp paired-end. Table 1 provides details of sequencing batch info. Library 977 (sample P0613) was first sequenced on a MiSeq V1 run, but due to the low number of bacterial reads obtained, this sample was later also included in a HiSeq2500 rapid sequencing run at a read length of 101 bp paired-end to achieve a comparable number of bacterial reads. We subsequently merged the reads from both sequencing run for this sample and treated these as one sample during data processing and comparisons.

### Data processing

As arterial plaque samples represent a host-associated metagenome, we mapped these reads against human reference genome (hg19) using bowtie 2-2.0.0 [17] with “very-sensitive” parameters to filter all human-like sequences from our samples. All unmapped reads (non-hg19) were extracted and aligned against non-redundant (nr) protein database (version 30.07.2012) [18] using BLASTX (ncbi-blast-2.2.25+; Max e-value 10e-3) [19]. After performing the BLASTX alignment, all output files of paired read sequences were imported and analyzed using the paired-end protocol of MEGAN5 [20].

### Taxonomic annotation

For processing the BLAST files by MEGAN5, we used parameter settings of “Min Score = 50”, “Top Percent = 10”, “Min Support = 25,” and “Minimum sequence complexity threshold = 0.44”. Some reads which did not have any match to the respective database were placed under a “No hit” node, and some reads that were originally assigned to a taxon that did not meet our selected threshold criterion were pushed back using the lowest common ancestor (LCA) algorithm to higher nodes where the threshold was met. After importing datasets in MEGAN, we obtained MEGAN-own “rma files” for each data mapped onto NCBI taxonomy based on our selected threshold. Further, only reads annotated as bacterial reads were extracted in new documents for each of the 22 samples and used for later analyses.

### Multiple metagenome comparison

All “rma files” were normalized to the smallest data set size without the not-assigned reads to allow inter-comparison of taxonomic abundances and to obtain comparative tree view for all samples. Additionally, “family” level taxonomic profile was used for hierarchical clustering with average linkage where Pearson correlation was used for clustering the families (rows) and Spearman correlation was used for clustering the datasets (columns) (Fig. 1). All computations were performed using R 3.0.2 [21]. Also, we performed principal coordinates analysis (PCoA) and unweighted pair group method with arithmetic mean (UPGMA) hierarchical clustering to compare taxonomic profiles of the samples at “species” level of NCBI taxonomy using MEGAN5 (Fig. 3).

### Rarefaction and diversity indices

We computed rarefaction curves from the normalized profile of 22 samples using the bacterial reads, showing the number of nodes that would be present in the analysis if based from 10 to 90 % of the reads. Further, “Shannon” and “Simpson” diversity indices were calculated for all samples (Table 2).

**Table 2:** Diversity indices

Sample	Total assigned bacterial species	ShannonH′ (loge)	Simpson 1-Lambda′
232	20	2.108	0.8319
233	129	1.753	0.575
234	49	2.816	0.9055
235	33	2.516	0.8869
236	18	1.942	0.8138
237	22	2.081	0.8451
238	361	3.524	0.918
239	15	1.941	0.8313
240	35	1.956	0.7783
241	28	2.306	0.863
242	14	1.898	0.8247
243	14	1.904	0.8263
48	16	1.516	0.6573
49	18	1.882	0.8169
50	18	1.718	0.7557
51	18	1.918	0.8018
52	32	1.995	0.7598
53	17	1.749	0.7501
54	12	1.541	0.7132
55	63	2.741	0.8437
56	20	1.205	0.4422
P0613_pooled	37	1.004	0.3896

Shannon-Wiener-diversity index (H) and Simpson’s diversity index (D) are reported for each sample

### Comparison of total biome: cases vs. controls

In addition to the above analyses, we merged the samples from the two patient groups, i.e., 15 symptomatic atherosclerosis patient samples (cases, Table 1) and the asymptomatic atherosclerotic samples from seven persons who died from causes not related to atherosclerotic disease (control, Table 1), to obtain a profile for the merged datasets in agreement with the view that up to the point of plaque instability, atherosclerosis pathogenesis is similar in symptomatic (unstable), and asymptomatic (stable) lesions. Only three samples (233, 238, and P0613) were not included in the merged samples (see the “Results” section).

### Functional annotation

Additionally, a functional analysis was performed on all samples using the SEED classification [22], based on the given BLASTX alignment and for only bacterial reads, using MEGAN5 as previously described [23]. In this classification scheme, genes are assigned to functional roles and genes with different functional roles are grouped into subsystems. The SEED classification can be represented as a rooted tree in which internal nodes represent different subsystems and where leaves represent functional roles.

To obtain a tentative pathway analysis, we performed an analysis based on Kyoto Encyclopedia for Genes and Genomes (KEGG) [24], where bacterial reads were mapped onto KEGG orthologous groups using MEGAN5. For such analysis, the MEGAN program matched each read to a KEGG orthology (KO) accession number, using the best hit to a reference sequence for which a KO accession number is known. The program reported the number of hits to each KEGG pathway. For such functional annotation using both SEED and KEGG, we first extracted all the reads that were mapped to bacteria in taxonomic annotations for all the datasets. Subsequently, we only processed these reads to obtain SEED and KEGG classifications to investigate metabolic or disease pathways of potential relevance to atherosclerosis

### Fluorescence in situ hybridization

Tissue samples were fixed with formalin and imbedded in paraffin. Samples were de-paraffinized with xylene, dehydrated, and washed. Oligonucleotide probes ACI208 (*Acidovorax* spp.), ClaR2 (*H. pylori*), and two negative control probes TM7305 (*Candidate division TM7*), PAR651 (*Paracoccus* spp.) (Sigma-Aldrich) were synthesized and labeled with Cy5 (Table 3). Fluorescence in situ hybridization (FISH) was performed according to a previously reported authoritative protocol [25]. Hybridization buffer was transferred onto the samples and followed by addition of individual gene probes (working solution 50 ng/uL). After applying FISH probes onto the tissue samples, slides were incubated in humidified environment at 46 °C for 3 h to hybridize the probe into target DNA. Different compositions of washing buffer were prepared corresponding to individual formamide concentrations. Then, slides were washed with preheated washing buffer at 48 °C (water bath) for 20 min, followed by snap dip in cold H_2_O and air-dried. Signals for each probe were observed under LSM 780 confocal laser scanning microscope (CLSM) (Carl Zeiss, Germany) equipped with GASP array detector and two PMT detectors for fluorescence signal detections and combination with T-PMT detector for transmitted images using Plan-Apochromat 40×/1.3NA oil objectives. Auto-fluorescence signals were checked using CLSM to avoid the biomass auto-fluorescence and determined the specific emission spectrum of each FISH probes.

**Table 3:** Fluorescence in situ hybridization (FISH) probe information

No.		Probe name	Sequence	Target
1	Target	ACI208	[Cyanine5] CGCGCAAGGCCTTGC	Acidovorax
2	Positive control	ClaR2	[Cyanine5] CGGGGTCTCTCCGTCTT	Clarithromycin-resistant *Helicobacter pylori* 23S rRNA
3	Negative control	PAR651	[cy5] ACC TCT CTC GAA CTC CAG	Genus *Paracoccus*
4	Negative control	TM7305	[cy5] GTC CCA GTC TGG CTG ATC	Subdivision 1 of candidate division TM7

### Data deposition

The sequence data obtained in this study have been deposited in the NCBI database under BioProject/BioSamples with accession number SRP040611.

## Additional files

Additional file 1: Figure S1. Taxonomic comparison of all DNA samples. Comparative analysis tree view for unstable plaque samples from seven patients with symptomatic atherosclerotic disease (cases, white) and five stable plaques from a control group (controls, gray) at “family” level of NCBI taxonomy. The scale shows the log value of reads assigned directly to a particular node. Some of the family hits that are likely to be of human origin are marked with black crosses. (EPS 923 kb) Additional file 2: Figure S2. Functional annotation using SEED subsystems. Multiple comparison tree view showing functional analyses using level 2 of SEED subsystems. The scale shows the log value of reads assigned directly to any particular subsystem. (EPS 1155 kb) Additional file 3: Figure S3. Functional annotation using KEGG orthologies. Multiple comparison tree view showing functional analyses using level 2 of KEGG orthologies. The scale shows the log value of reads assigned directly to any particular subsystem. (EPS 487 kb)
